# Supplementary material for: Trichoderma harzianum Cellulase Gene thph2 Affects Trichoderma Root Colonization and Induces Resistance to Southern Leaf Blight in Maize
Source: J Fungi (Basel). 2023 Dec 4;9(12):1168. doi: 10.3390/jof9121168 (PMC10744625; doi:10.3390/jof9121168)
Supplement: Supplementary file 1 [file jof-09-01168-s001.zip › jof-2644924-supplementary.pdf]

Table S1. Primers used in this study.

Primer name	Sequence (5'-3'; restriction sites underlined)	Description
18s-F	CCTGCGGCTTAATTGACTC	qRT-PCR primer for 18s as reference
18s-R	GTTAGCAGGCTGAGGTCTCG	
AOS-F	TTCCTCCGCTACGACACATT	qRT-PCR primer for *ZmAOS*
AOS-R	CCGAGGGTGAGAGAGAAGTC	
HPL-F	CCACACACGGGAGATAGCTA	qRT-PCR primer for *ZmHPL*
HPL-R	GAAGAACTCGTCCTGGCTCT	
LOX5-F	TCGCGTCTACCGTTACGACTACTA	qRT-PCR primer for *LOX5*
LOX5-R	TTCAGGTTCAGCAGGAAAAGC	
OPR1-F	CGTATGGGAGGCTGTTCTTG	qRT-PCR primer for *OPR1*
OPR1-R	AGCGGTCGTATTTGTTGAGTG	
ZmPR1-F	GGCGAGAGCCCCTACTAGAC	qRT-PCR primer for *ZmPR1*
ZmPR1-R	AAATCGCCTGCATGGTTTTA	
ZmPR5-F	GTCATCGACGGCTACAACCT	qRT-PCR primer for *ZmPR5*
ZmPR5-R	CACGGGCAGAAGGTGACT	
PAL-F	GAAGCTCATGTCGTCCACCTA	qRT-PCR primer for *PAL*
PAL-R	GTTCATGGTCAGCACCTTCTT	
